# Phyto-Mediated Copper Oxide Nanoparticles for Antibacterial, Antioxidant and Photocatalytic Performances

**DOI:** 10.3389/fbioe.2022.820218

**Published:** 2022-02-16

**Authors:** Kenneth Ssekatawa, Denis K. Byarugaba, Martin Kamilo Angwe, Eddie M. Wampande, Francis Ejobi, Edward Nxumalo, Malik Maaza, Juliet Sackey, John Baptist Kirabira

**Affiliations:** ^1^ College of Veterinary Medicine Animal Resources and Biosecurity, Makerere University, Kampala, Uganda; ^2^ Institute for Nanotechnology and Water Sustainability, College of Science, Engineering and Technology, Florida Science Campus, University of South Africa, Pretoria, South Africa; ^3^ Nanosciences African Network (NANOAFNET), iThemba LABS-National Research Foundation, Somerset West, South Africa; ^4^ UNESCO-UNISA Africa Chair in Nanosciences/Nanotechnology, College of Graduate Studies, University of South Africa (UNISA), Pretoria, South Africa; ^5^ Africa Center of Excellence in Materials, Product Development and Nanotechnology, College of Engineering, Design, Art and Technology, Makerere University, Kampala, Uganda

**Keywords:** antibiotic resistance, photocatalysis, antioxidant activity, green synthesis of nanoparticles, copper oxide nanoparticles, *Prunus africana*, *Camellia sinensis*

## Abstract

The greatest challenge of the current generation and generations to come is antimicrobial resistance, as different pathogenic bacteria have continuously evolved to become resistant to even the most recently synthesized antibiotics such as carbapenems. Resistance to carbapenems limits the therapeutic options of MDR infections as they are the only safe and effective drugs recommended to treat such infections. This scenario has complicated treatment outcomes, even to the commonest bacterial infections. Repeated attempts to develop other approaches have been made. The most promising novel therapeutic option is the use of nanomaterials as antimicrobial agents. Thus, this study examined the efficacy of *Camellia sinensis* extract (CSE) and *Prunus africana* bark extract (PAE) green synthesized Copper oxide nanoparticles (CuONPs) against carbapenem-resistant bacteria. Furthermore, the photocatalytic and antioxidant activities of CuONPs were evaluated to determine the potential of using them in a wide range of applications. CuONPs were biosynthesized by CSE and PAE. UV vis spectroscopy, X-ray Diffraction (XRD), Dynamic light scattering (DLS), Fourier Transform Infrared spectroscopy (FTIR), and Scanning Electron Microscopy (SEM) were used to characterize the nanoparticles. CuONPs susceptibility tests were carried out by the agar well diffusion method. The photocatalytic and antioxidant activities of the CuONPs were determined by the methylene blue and DPPH free radical scavenging assays, respectively. UV vis absorbance spectra registered surface plasmon resonance peaks between 272 and 286 nm, confirming the presence of CuONPs. The XRD array had nine strong peaks at 2θ values typical of CuONPs. FTIR spectra exhibited bands associated with organic functional groups confirming capping and functionalization of the CuONPs by the phytochemicals. DLS analysis registered a net zeta potential of +12.5 mV. SEM analysis revealed that the nanoparticles were spherical and clustered with a mean diameter of 6 nm. Phytosynthesized CuONPs exhibited the highest growth suppression zones of 30 mm with MIC ranging from 30 to 125 μg/ml against MDR bacteria. Furthermore, the CuONPs achieved a methylene blue dye photocatalysis degradation efficiency of 85.5% and a free radical scavenging activity of 28.8%. PAE and CSE successfully bio-reduced copper ions to the nanoscale level with potent antimicrobial, photocatalysis, and antioxidant activities.

## Background

Antibiotics have been the nucleus of chemotherapy since their discovery and introduction into the healthcare system in the 1940s. They are used routinely not only to treat fatal bacterial infections but also to prevent infections in patients with compromised immune systems and to enhance growth in livestock (Centre for Disease Dynami, 2015; Laxminarayan et al., 2015). The bigger the quantity of antibiotics used, the higher the probability of evolution of antibiotics resistant bacteria in the context of natural selection at the bacterial level; therefore, antibiotic resistance is directly associated with antibiotics overuse and misuse (Laxminarayan et al., 2015). As a result, once familiar antibiotics susceptible bacterial infections are hard to treat, increasing expenses of healthcare facilities and patient mortality rate as last resort antibiotics are very costly and unavailable to the rural poor. Furthermore, resistance to last-resort antibiotics used in treating multidrug-resistant (MDR) infections has been reported worldwide (Centre for Disease Dynami, 2015). For example, several studies have reported a high prevalence of carbapenem resistance in Uganda (Ssekatawa et al., 2018; Ssekatawa et al., 2021a; Ssekatawa et al., 2021b), yet carbapenems are a class of antibiotics earmarked to treat MDR gram-negative bacterial infections. Thus, resistance to carbapenems limits the treatment options for MDR infections. Therefore, there is a need for alternative approaches to complement and reduce the overuse of antibiotics.

Due to nanoparticles hypothesized superior properties, nanotechnology has been successfully embraced by researchers in biomedical sciences. Organic nanoparticles such as chitosan have captured the centre stage of oral, nasal, and intravenous drug delivery (Casettari and Illum, 2014; Islam and Ferro, 2016). Among the metal nanoparticles, silver nanoparticles (AgNPs) are at the heart of research owing to their potent antimicrobial activity against MDR bacterial, viral and fungal infections (Morones et al., 2005; Jain et al., 2009) and anti-cancer activities (Gopinath et al., 2008). Furthermore, copper nanoparticles (CuNPs) or copper oxide nanoparticles (CuONPs) have received significant attention as alternatives to AgNPs in the medical field due to their unquestionable broad-spectrum bactericidal activity, in addition to their low cost, physicochemical stability, biocompatibility properties, and compatibility with other materials to form polymers (Karthik and Geetha, 2013; Potbhare et al., 2019). Therefore, CuNPs present a possible alternative to antibiotics.

Furthermore, synthetic dyes are discharged into the environment from the food processing, paper, plastic, tannery, and cosmetics industries. Heavy metals from mining industries also find their way into nature. A high concentration of pollutants in the environment leads to adverse effects on all life forms. For example, the dyes and heavy metal contaminants cause cancer, allergies, severe effect on the mammalian central nervous system, and kidney and liver damage (Chandraker et al., 2020). Removing industrial wastes before being discharged into the environment is necessary to thwart heavy metals and dye-related complications. Several approaches such as photocatalysis, nanosensor, organic drugs, and coagulation/flocculation have been employed to remove pollutants from the environment (Tahir et al., 2019a; Tahir et al., 2019b). Research on photocatalytic degradation of dyes and chemical contaminants by nanoparticles under sunlight irradiation is of primary interest. Data on photocatalytic degradation of pollutants CuONPs is scanty. However, biosynthesized copper nanoparticles have been reported to be semiconductor materials; therefore, electrons migrate from the valence band to the conduction band under sunlight irradiation, thereby degrading dyes and chemicals (Tahir et al., 2018a; Tahir et al., 2019b; Chandraker et al., 2020). Indeed, preliminary studies reported potent photocatalytic degradation of dyes by copper nanoparticles and other nanomaterials (Tahir et al., 2018b; Chandraker et al., 2020; Mali et al., 2020). Additionally, reactive oxygen species (ROS) are intrinsic by-products of normal body metabolism and are firmly regulated by antioxidants. However, ROS can cause impairment to lipids, proteins, nucleic acids when generated in excess or when antioxidants are inadequate. This state of oxidative stress is associated with several degenerative disorders. The harmful effects of ROS can effectively be lowered by reinstating the balance between generation and elimination of ROS through taking of antioxidants (Taverne et al., 2013). On top of the list of the potential candidates to be administered as antioxidants to combat oxidative stress are CuNPs, as they have exhibited promising free radical scavenging activities in *in vitro* experiments (Rajeshkumar et al., 2019; Das et al., 2020; Wu et al., 2020). With this background, copper nanoparticles possess a wide variety of applications.

As metal nanoparticles, CuONPs can be fabricated using conventional methods categorized as physical and chemical methods. However, the traditional methods possess limitations that cannot be easily mitigated. Synthesis of nanoparticles using physical processes such as ball milling, evaporation/condensation, radiolysis, and laser ablation is complicated and requires expensive facilities and excessive energy, therefore unpopular in countries with limited resources (Thakkar et al., 2010). Whereas the chemical approach employs expensive hazardous chemicals to reduce metal ions to nanoscale metal clusters that are most likely contaminated with toxic chemicals hence exhibiting nonselective toxicity to both the target bacteria and the host cells (Kawata et al., 2009; Tahir et al., 2013). Additionally, metal nanoparticles synthesized by physical and chemical means require post-fabrication modification to become operational (Castro et al., 2014).

To mitigate those challenges, the fabrication of nanoparticles using a cheaper, user, and environmentally friendly approach known as green synthesis has gained considerable popularity among many researchers. Green synthesis/green chemistry is a chemical-free method that involves the use of extracts with antioxidant activity. The extracts are obtained from living organisms such as plants, bacteria, and fungi; thus, the term green synthesis is interchangeably used with biosynthesis. Plant crude extracts hold a variety of secondary metabolites such as polyphenols, alkaloids, flavonoids, and terpenoids with antioxidant activity (Elmusa et al., 2021). The antioxidant properties of the extracts potentiate them to function as electron acceptors, thereby reducing metal salts to nanoscale by scavenging electrons from them (Kharat and Mendhulkar, 2016; Fafal et al., 2017). Thus, several researchers have reported the use of phytochemicals with antioxidant activity such as green tea, lichen *Parmotrema praesorediosum*, *Musa AAA*, *Jatropha curcas*, *Scutellaria baicalensis*, *Pulicaria glutinosa* extracts, among others as well as *Fusarium oxysporum* strains of fungi to reduce metal ions to metal nanoparticles (Durán, 2005; Khan et al., 2013; Huang et al., 2014; Mie et al., 2014; Shaik et al., 2018; Ituen et al., 2019; Tettey and Shin, 2019; Wagutu et al., 2019). The plant extracts decompose metal ions to their nanoscale form and subsequently cap them to prevent agglomeration, making them physiologically compatible (Castro et al., 2014). Furthermore, green synthesized nanoparticles are functionalized by the compounds found in the extracts used in the bio-reduction of the metal ions. Thus, the functionalized nanoparticles can easily be conjugated to polymers (silica, chitosan, nucleic acids, and proteins) and drugs through bonding with the organic functional groups. Due to the ability to readily form H-bonds between the organic functional groups on the nanoparticles and the molecule to be delivered, biosynthesized nanoparticles are good candidates for designing nano delivery systems. Furthermore, conjugating functionalized nanoparticles with antibacterial activity to antibiotics re-potentiates antibiotics against resistant bacteria (Arul Selvaraj et al., 2019; Ssekatawa et al., 2020). Therefore, green synthesis has made it possible to produce functionalized nanoparticles that can be used to design therapeutic drug-nanomaterial complexes with enhanced antimicrobial activity in environments with limited resources.

The size and shape of the biosynthesized inorganic nanoparticles are influenced by several reaction parameters such as the concentration of inorganic salt, pH of the reaction mixture, the concentration of plant extract, reaction temperature, and reaction duration. Thus, the physicochemical properties of the green synthesized nanoparticles rely on the optimization of the reaction parameters. Therefore, determining the conditions suitable for the biosynthesis of inorganic nanoparticles is achieved through varying the parameters mentioned above. Furthermore, the essential phytochemicals with antioxidant activity in the plant extract affect the physicochemical properties of the biosynthesized nanoparticles, and the antioxidants present vary from species to species (Usmani et al., 2018; Akintelu et al., 2020). *Camellia sinensis* extract (CSE) and *Prunus africana* extract (PAE) are rich in phytochemicals with strong free radical scavenging activity and medical importance. The main classes of phytochemicals in PAE include pentacyclic triterpenoids, phytosterols and ferulic acid esters. The three phytochemical types possess potent antioxidant activity (Bin Sayeed et al., 2016; Ghante and Jamkhande, 2019) and have demonstrated anti-prostate cancer and benign prostate hyperplasia (BPH) activities; thus, PAE was patented in France as anti-prostate cancer and BPH agent (Schleich et al., 2006). Whereas fresh *Camellia sinesis* leaves processed into green tea, hold bioactive polyphenols called catechins with potent antioxidant activity (Botten et al., 2015). As such, green tea is widely used to manage chronic oxidative stress-related ailments (Hajiaghaalipour et al., 2016; Takahashi et al., 2018). With that background, PAE and CSE can biosynthesize biocompatible, stabilized, functionalized inorganic nanoparticles. Ssekatawa et al. (Ssekatawa et al., 2021c) evaluated the influence of parameters such as reaction time, plant extract concentration, and plant species on the physicochemical properties of *Camellia sinensis* and *Prunus africana* biosynthesized silver nanoparticles. Findings revealed that both plant extracts biosynthesized small-sized spherical nanoparticles at neutral pH and room temperature (25–26°C). The reaction rate, size, and agglomeration of nanoparticles increased with a rise in plant extract concentration and time. And *Camellia sinensis* extract synthesized smaller nanoparticles. However, the impact of pH and temperature on the properties of nanoparticles were never investigated. Thus, this study evaluated the effect of temperature and pH on the properties of *Camellia sinensis and Prunus africana* green synthesized CuONPs. For application purposes, the efficacy of the biosynthesized CuONPs against methicillin-resistant *Staphylococcus aureus*, carbapenem-resistant (CR) *E. coli*, and *K. pneumoniae* was investigated. Additionally, the photocatalytic and antioxidant activities of the biosynthesized nanoparticles were assessed. Furthermore, this study provides a foundation for further exploitation of the diverse Ugandan medicinal flora for inorganic nanoparticle biosynthesis that could be used for several applications.

## Materials and Methods

### Site Description and Source of Materials

This study was carried out at the College of Veterinary Medicine, Animal Resources and Biosecurity (CoVAB), Makerere University, Materials Research Department (MRD) iThemba LABs, Cape Town and Nanotechnology Unit, University of South Africa (UNISA). Processing of plant material, phytochemical extraction, and green synthesis of CuONPs were conducted from the Pharmacology laboratory, CoVAB, and MRD. Physiochemical characterization of CuONPs was executed from MRD and the Nanotechnology Unit, UNISA, while CuONPs bactericide assays were performed from the General Microbiology Laboratory, CoVAB. Confirmation of the identity of *Prunus africana* was done from the department of Botany, Makerere University. Analytical grade Copper II nitrate (Cu(NO_3_)2•3H_2_O) was obtained from Sigma Aldrich, United States. Fresh *Camellia sinensis* branches with leaves were purchased from Igara Tea Estates, Bushenyi, while *Prunus africana* bark and branches with leaves were acquired from Maramagambo forest, which covers the southern part of Queen Elizabeth National Park located in Bushenyi district, Uganda, with approval of National Forestry Authority. No further approvals were required to use the plant materials in the study.

### Preparation of Plant Materials for Extraction

Fresh tea leaves were purchased from Igara Tea Estates Bushenyi, while fresh bark and leaves of *Prunus africana* were obtained from Maramagambo forest, Bushenyi district. Using freshly collected branches with leaves and flowers, the identity of the plants was ascertained by Mrs Carol Kauma, a botanist at the school of botany Makerere University. Voucher specimens were deposited at the Makerere University Herbarium, College of Natural Sciences with accession numbers MHU51015 for *Prunus africana* and MHU51016 for *Camellia sinensis* (L.) Kuntze. Plant materials were cleaned thoroughly under running tap water to remove lichen and dirt and then transferred to an evaporator at 30°C to remove all the moisture. The plant materials of *Camellia sinensis* and *Prunus africana* were dried separately under darkness at room temperature in a drying cabinet for 20 days. The dried plant materials were sliced into small pieces and milled to fine powder by an electric grinder.

### Extraction of Phytochemicals

For each powder type, 500 g were mixed thoroughly with 2 L of chilled sterile distilled deionized water in dark extraction bottles and left to stand for 120 h but with occasional vortexing to ensure uniform phytochemical extraction. The aqueous mixtures were strained through Whatman filters size 1 with the aid of a vacuum suck pump and concentrated under vacuum using a Heldolph Laborata 4000-efficient rotary evaporator at 40°C for 12 h. The concentrates were transferred to evaporation pans and dried to a powder by a hot air oven set at 40°C. The obtained dry powders from each plant extract (PE) were stored in dark containers at 4°C until further use.

### Green Synthesis of Copper Nanoparticles Using Plant Extracts

Copper nanoparticles were biosynthesized by adding 1 ml of *Camellia sinensis* extract (CSE) to a conical flask containing 49 ml of an aqueous solution prepared by dissolving 120.8 mg of Copper II Nitrate in 1,000 ml of distilled deionized water (0.5 mM). The pH value of the Copper II Nitrate and CSE mixture was recorded. To determine the effect of pH on the green synthesis of copper nanoparticles, three more copper II nitrate (0.5 mM) and CSE mixtures labelled pH 2, 7, and 9 were prepared as described above, followed by adjusting the pH to 2 7, and 9, respectively. The same procedure was repeated for *Prunus africana* extract (PAE). This procedure was duplicated to generate two setups.

### Setup 1

The eight conical flasks were fixed with cooling condensers. The reaction mixture was left to stand in a revolving (5 rpm) water bath at 80°C for 8 h, then left to cool down at room temperature and centrifuged for 30 min at 9,000 rpm. The sediments obtained from the different treatments were washed numerous times with distilled deionized water, after which the final residues produced were dried at 80°C in an oven for 12 h, then transferred into 1.5 ml Eppendorf tubes and stored at room temperature (25–26°C).

### Setup 2

The eight conical flasks were sealed in aluminium foil and left to stand in a dark chamber for 24 h at room temperature (25–26°C), after which, they were transferred into a hot air oven set at 120°C to evaporate off all the liquid content. The burnt brown substances were scraped off the bottom of the beakers and transferred into 1.5 ml Eppendorf tubes using a spatula and stored at room temperature (25–26°C).

## Characterization of Copper Oxide Nanoparticles

### UV–Vis Spectroscopy

UV-Vis spectrophotometry analysis was carried out using 6715 UV Vis Spectrophotometer, Jenway. Briefly, samples (1 ml) were obtained from setup one after the 8 h and setup two after 24 h and dispensed into different cuvettes. To obtain the optical properties of the green synthesized copper nanoparticles, each sample was examined by the UV Vis spectroscope scanning at a resolution of 1 nm between 200 and 700 nm ranges.

### X-Ray Diffraction Analysis

To enhance the crystallinity of the biosynthesized copper oxide nanoparticle, about 1 g of the powder was annealed at 500°C for 2 h in a furnace and then examined by powder X-ray diffraction (XRD) using BRUKER AXS diffractometer, D8 Advance (Germany) fitted with Cu-Ka radiation (*λ*Kα_1_ = 1.5406 Å) from 2θ = 0.5°–130°, with increment Δ2ϑ: (0.034°), a voltage of 40 kV, current of 40 mA, power of 1.6 kW and counting time of 0.5 s/step. To evaluate the effect of storage time on the stability and viability of the nanoparticles, XRD analysis was repeated after 6 months. The resultant data was visualized in OriginPro 8.5, and the XRD diffraction patterns were validated using standard Copper oxide nanoparticles 2θ values from the International Center for Diffraction Data (ICDD) database. The average crystallite size was computed using Debye-Scherrer’s equation given below;

$$d=\frac{0.9\lambda }{\beta \;cos\theta \;}$$ (1)

Where d is the diameter or average crystallite size, β is full peak width at half maximum (FWHM), λ is the X-ray wavelength used (1.5406 Å), θ is the 2θ angle in peak.

### Fourier Transform Infrared Spectroscopy (FTIR)

The organic functional groups of phytochemicals in PEs used in green synthesis and stabilization of Copper Oxide nanoparticles were identified by Fourier transform Infrared spectroscopy (PerkinElmer Spectrum RX I Fourier transform IR system) with a wavenumber frequency ranging from 4,000 to 400 cm^−1^ and a resolution of 4cm^−1^. Two milligrams (2 mg) of Copper oxide nanoparticles and 2 g of Potassium bromide (KBr) were dehydrated at 200°C in an air oven overnight. The 2 mg of dried Copper oxide nanoparticles were mixed thoroughly with 100 mg of anhydrous KBr to obtain a homogenous mixture and then compressed to obtain translucent circular disc-shaped pellets. The pallets were scanned through 4,000–400 cm^−1^ wavenumber range for at least 64 scans per sample. A KBr pellet was used as a control. FTIR analysis was repeated after 6 months of storage to evaluate the effect of storage time on the organic phytochemicals that cap, stabilize and functionalize the nanoparticles.

### Dynamic Light Scattering and Zeta Potential

To determine the average size distribution and zeta potential of copper oxide nanoparticles, a zeta sizer Nano ZS Malvern Panalytical was used. The DLS package was used to evaluate the average nanoparticle size distribution as follows; 100 μg/ml was sonicated for 5 min to deagglomerate and suspend the nanoparticles in Millipore water, and the dynamic particle sizes were estimated by adding 1 µL of the sonicated nanoparticle in 1 ml of Millipore water in zeta sizer cuvette. This was followed by examining the nanoparticle suspension by a DLS analyzer 60 times per scan for three scans. The zeta potential of copper oxide nanoparticles was estimated using electrophoretic light scattering technology. A concentration of 1 mg/ml of nanoparticle suspension was made in Millipore water in a 900 μL zeta sizer disposable cell. The suspension was analyzed 60 times per scan for three scans.

### Scanning Electron Microscopy and Elemental Analysis

The particle size distribution and microstructure of the green synthesized CuONPs were studied by the field emission scanning electron microscopy (FESEM) coupled with Energy Dispersive X-ray (EDX)spectroscopy, Carl Zeiss Sigma 300 VP model operating at 5.0 kV. In brief, 1 mg of CuONPs was spread onto a conducting carbon tape to form a thin film. The thin film of CuONPs was carbon-coated, and images were taken at different magnifications. Size distribution of nanoparticles was estimated from the FESEM images using ImageJ software. The EDX spectra were obtained at an acceleration voltage of 20 kV and collected for 19s. Mapping was completed using pseudo-colours to represent the two-dimensional spatial distribution of energy emissions of the chemical elements present in the samples.

### Copper Oxide Nanoparticles Susceptibility Bioassay

Antibacterial activity of the biosynthesized CuONPs was evaluated using the agar well diffusion method. Carbapenem-resistant *E. coli* ATCC 96522 and *K. pneumoniae* NTCT 9633 and Methicillin-resistant *Staphylococcus aureus* ATCC 33592 obtained from the Microbiology department College of Health Sciences, Makerere University were used. The bacteria isolates at 0.5 McFarland’s units were inoculated on separate sterile Muller Hinton Agar plates with sterile inoculating loops. Wells labelled negative control (−ve), positive control (+ve), 50-CuONPs, 100-CuONPs and 250-CuONPs were cut into the agar plates containing the bacterial lawns. CuONP solutions were prepared at 50, 100 and 250 mg/ml concentrations in DMSO. Fifty microliters (50 μL) of each CuONPs solution was dispensed into their corresponding wells. Imipenem (10 μg and ampicillin (30 μg) discs were used as the standard drugs, while distilled water in DMSO pipetted in the well labelled −ve was used as the negative control. The setup was left to set for 1 h, then incubated aerobically for 24 h at 37°C and checked for antibacterial activity. The clearance zone for the positive control (+ve), negative control (−ve), and CuONPs were recorded in millimetres. The experiment was repeated three times to get the average zone of inhibition (ZOI). Furthermore, to assess the reusability of the biosynthesized CuONPs after storage, the agar well diffusion assay was repeated after 6 months.

### Antimicrobial Assay by Broth Serial Dilution Method

Broth serial dilution method was used to determine the minimum inhibitory concentration (MIC) and minimum bactericide concentration (MBC) of phytosynthesized CuONPs. Briefly, 1 mg/ml of CSE-CuONPs was constituted using distilled deionized water in a sterile falcon tube. Then 50 μL of sterile tryptic soy broth (TSB) was pipetted into the ten wells of a microtiter plate labelled *E. coli.* This step was replicated in the second and third rows of the microtiter plate, and the rows were labelled *K. pneumoniae* and *S. aureus*, respectively. A two-fold serial dilution was carried out by pipetting 50 μL of the CuONPs solution into the first wells of each row. The tryptic soy broth and CuONP solution were thoroughly mixed into a homogenous solution. This was followed by the transfer of 50 μL of the mixture from the first well to the second well of each row. This step was repeated until the eighth well. This was followed by dispensing 0.5 μL of fresh bacterial culture of *E. coli*, *K. pneumoniae* and *S. aureus* at 0.5 McFarland’s turbidity standard into the corresponding wells containing CuONPs diluents and the 9^th^ wells (positive control). The 10^th^ wells had only broth; thus, they served as the negative control. The plate was incubated aerobically for 24 h at 37°C, followed by plating the samples from each well onto MHA. The plates were left to stand all night at 37°C under aerobic conditions. MIC was taken as the concentration of CuONPs with the minimum growth inhibition. MBC was determined by further performing a tenfold serial dilution for the dilutions that didn’t show growth under MIC determination experiments. The diluents were further plated on MHA plates. The plates were incubated at 37°C for 24 h and then observed for growth. MBC was taken as the concentration of the CuONPs in the well before the one with growth. The above procedure was repeated for PAE-CuONPs.

### Photocatalytic Activity of CuONPs

The photocatalysis potential of the biosynthesized CuONPs was evaluated by the degradation of an aqueous solution of methylene blue (MB) under sunlight treatment using adjusted methods previously described by Chandraker et al (Chandraker et al., 2020). Briefly, 100 μg/mL MB solution was prepared by adding 1 mg to 100 ml of deionized water. Ten milligrams (10 mg) CSE-CuONPs were mixed thoroughly with the 100 ml of 100 μg/mL MB solution and pH adjusted to 9.0 under dim light at room temperature (25°C ± 2). A second setup was prepared for PAE-CuONPs photocatalysis analysis. A control experiment was setup where no CuONPs were added. The solutions were left to stand under direct sunlight. The dye degradation rate was estimated by measuring absorbance at 614 nm using a UV-1800 Shimadzu UV vis spectrophotometer at 0, 5, 30, 60, 90, 120, 150, and 180 min. For each reading, about 3 ml of the suspension from each setup was taken and centrifuged to remove the suspended CuONPs. All the setups were duplicated to obtain average absorbance for each time interval. The photocatalytic degradation efficiency was assessed based on the following formulae. The entire experiment was repeated, but the flasks were sealed in aluminium foil and kept under darkness.

$$Ct=\frac{absorbance\;of\;the\;test}{absorbance\;of\;the\;control}X\;Co$$ (2)

$$\%\;degradation=\frac{Co-Ct}{Co}\;X\;100$$ (3)

Where C_t_ is the concentration at time t and (Co) is the initial concentration of MB.

### Antioxidant Activity

The 2,2-diphenylpicrylhydrazyl (DPPH) free radical scavenging assay was employed to evaluate the antioxidant activity of the phytosynthesized CuONPs, CSE, and PAE. This assay was carried out modifying methods previously outlined by Wu et al. (Wu et al., 2020). Briefly, a stock solution of 1 mg/ml concentration was prepared by dissolving 10 mg of the biosynthesized CuONPs and the plant extracts in 10 ml of methanol. The stock solutions were used to prepare solutions of 50, 100, 150, 200, 250, and 300 μg/ml concentrations. About 0.5 ml of each antioxidant solution was pipetted to 3 ml of 0.5 mM DPPH in methanol solution in a 15 ml falcon tube. A parallel experiment was conducted using ascorbic acid as the standard antioxidant. The DPPH solution was not treated with any test solutions (CuONPs, CSE, and PAE) for the control experiment. The reaction mixtures were left to stand under darkness at room temperature (25°C ± 2). After 30 min, a change in colour from violet to yellow was observed. The absorbance of the reaction mixture was then recorded at 517 nm using a UV-1800 Shimadzu UV vis spectrophotometer. The DPPH percentage inhibition was computed using the following formula;

$$\%\;scavenging\;activity=\frac{\left(absorbance\;of\;the\;negative\;control-absorbance\;of\;the\;test\right)\;}{absorbance\;of\;the\;negative\;control}\;X\;100$$ (4)

### Data Analysis

Data analysis was done using IBM SPSS Statistics Version 25. Comparison of MIC, MBC, mean zone bacterial growth inhibition, photocatalysis percentage degradation efficiency, percentage scavenging activity for CSE-CuONPs and PAE-CuONPS was done by one-way ANOVA. A *p*-value of ≤0.05 specified considerable statistical variance.

## Results

### Optical Properties of Colloidal Green Synthesized Copper Nanoparticles

**FIGURE 1:**
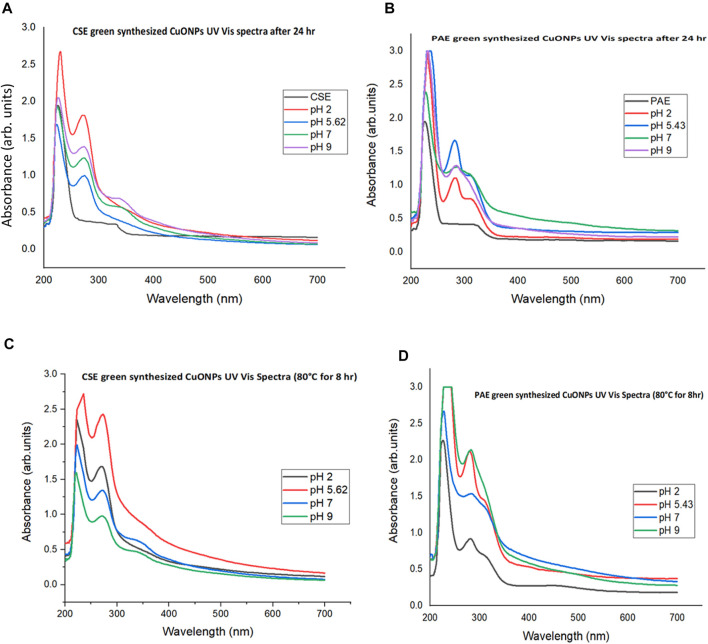
UV Vis absorbance spectra for green synthesis of CuONPs at different pH Values. **(A)**: UV Vis spectra for CSE biosynthesized CuONPs at room temperature for 24 h, **(B)**: PAE biosynthesized CuONPs at room temperature for 24 h, **(C)**: CSE biosynthesized CuONPs at 80°C for 8 h, and **(D)**: PAE biosynthesized CuONPs at 80°C for 8 h.

UV Vis absorption spectra for colloidal CuONPs-plant extract mixture at different pH were recorded to demonstrate how pH influences the green synthesis of copper nanoparticles. The pH values of CSE-Copper nitrate and PAE-Copper nitrate mixtures were 5.62 and 5.43, respectively, Table 1. UV Vis absorption spectra registered narrow absorbance peaks ranging from 272 to 275 nm and 281–286 nm for CSE, and PAE green synthesized nanoparticles, respectively. High-intensity peaks were recorded for CSE, and PAE biosynthesized CuONPs in low pH and slightly high pH media, respectively. Furthermore, peak intensities revealed that setup one, which involved incubation at 80°C for 8 h, was more efficient than the setup that was left to stand at room temperature (25–26°C) for 24 h, Figure 1; Supplementary Table S1. All the absorbance spectra registered peaks ranging from 225 to 230 nm, Figure 1.

**TABLE 1:** 2θ angles and their corresponding Miller’s incident, peak intensity, and average crystal size of CSE and PAE phytosynthesized nanoparticles.

PAE-CuO NPs					CSE-CuO NPs				
2θ (Deg)	Int (a.u)	Miller’s indices	FWHM (Rad)	Average particle size (nm)	2θ (Deg)	Int (a.u)	Miller’s indices	FWHM (Rad)	Average particle size (nm)
32.5	236	(110)	0.00682	24	35.5	897	(002)	0.00811	21
35.5	1,746	(002)	0.00682	25	38.7	829	(111)	0.00811	22
38.7	1,653	(111)	0.00682	26	48.7	263	(202ˉ)	0.00811	26
48.9	469	(202ˉ)	0.00682	31	53.5	113	(020)	0.00811	29
53.5	167	(020)	0.00682	34	58.3	113	(202)	0.00811	33
58.3	248	(202)	0.00682	39	61.5	198	(113ˉ)	0.00811	36
61.5	349	(113ˉ)	0.00682	43	65.9	197	(022)	0.00811	42
66.3	320	(311ˉ)	0.00682	51	68.1	265	(220)	0.00811	46
68.1	326	(220)	0.00682	55	72.4	123	(311)	0.00811	57
72.5	133	(311)	0.00682	68	75.2	113	(222ˉ)	0.00811	67
75.3	217	(222ˉ)	0.00682	80	—	—	—	—	—
Mean size	—	—	—	43	—	—	—	—	38

### X-Ray Diffraction Analysis

**FIGURE 2:**
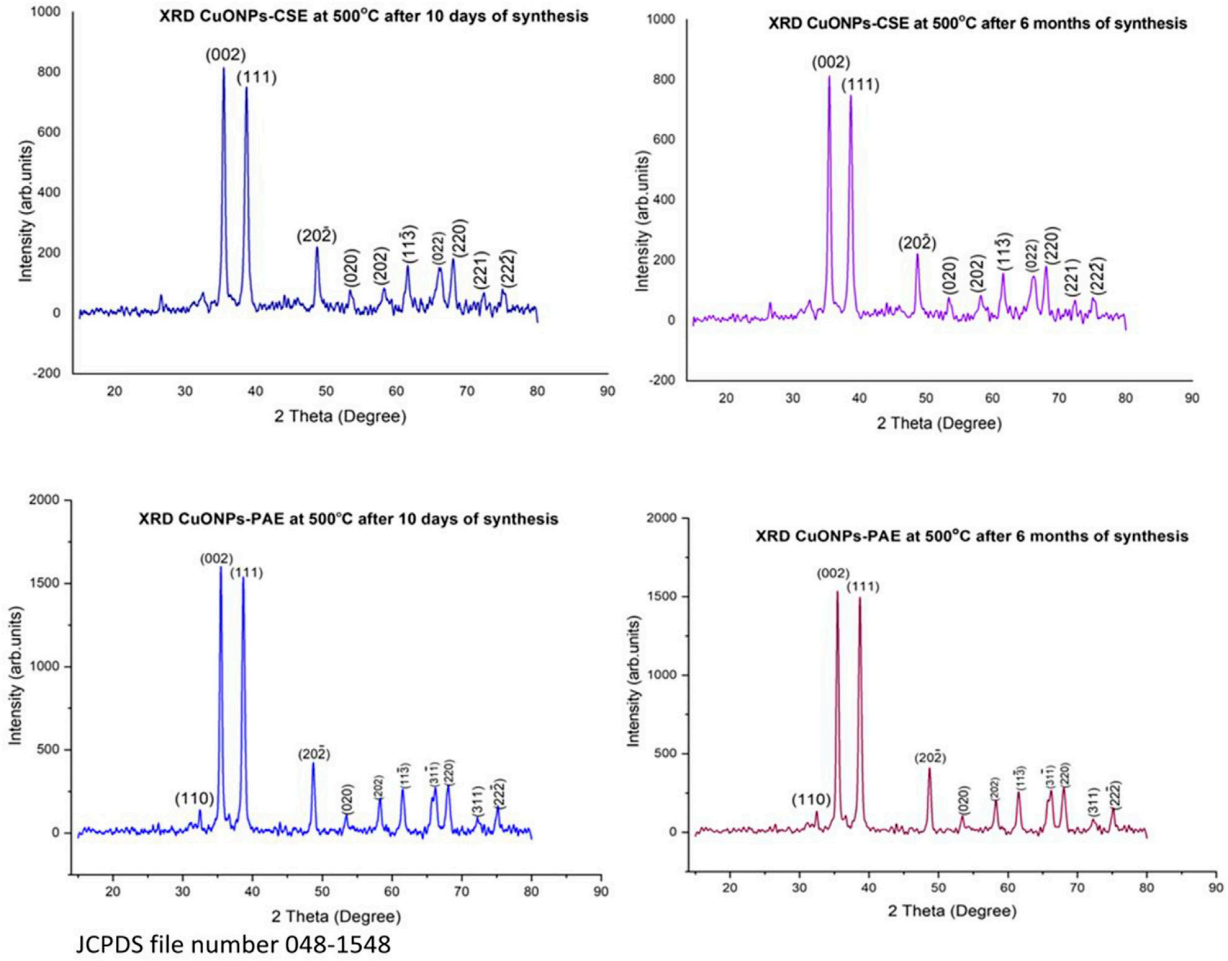
XRD pattern for phytosynthesized copper oxide nanoparticles.

X-Ray Diffraction patterns of the biosynthesized CuO NPs generated nine-strong peaks for both PAE and CSE. Bragg peaks situated at 2θ values of 35.5°, 38.7°, 48.9°, 53.5°, 58.3°, 61.5°, 68.1°, 72.5° and 75.3° for both PAE and CSE mediated CuONPs were registered. PAE mediated CuONPs had two unique extra Bragg peaks observed at 2θ angles of 32.5° and 66.3° while CSE fabricated CuONPs expressed one exceptional Bragg peak at 2θ angle of 65.9°. Average crystallite size calculated from XRD pattern using Debye-Scherrer’s formula ranged from 24 to 80 nm with a mean of 43 nm, and 21–67 nm with a mean of 38 nm for PAE and CSE biosynthesized nanoparticles, respectively, Table; Figure 2. To assess the stability of the biosynthesized, XRD analysis was conducted after 6 months of storage and exhibited identical patterns to those of freshly prepared CuONPs, Figure 2.

### Fourier Transform Infrared Spectroscopy Analysis

**FIGURE 3:**
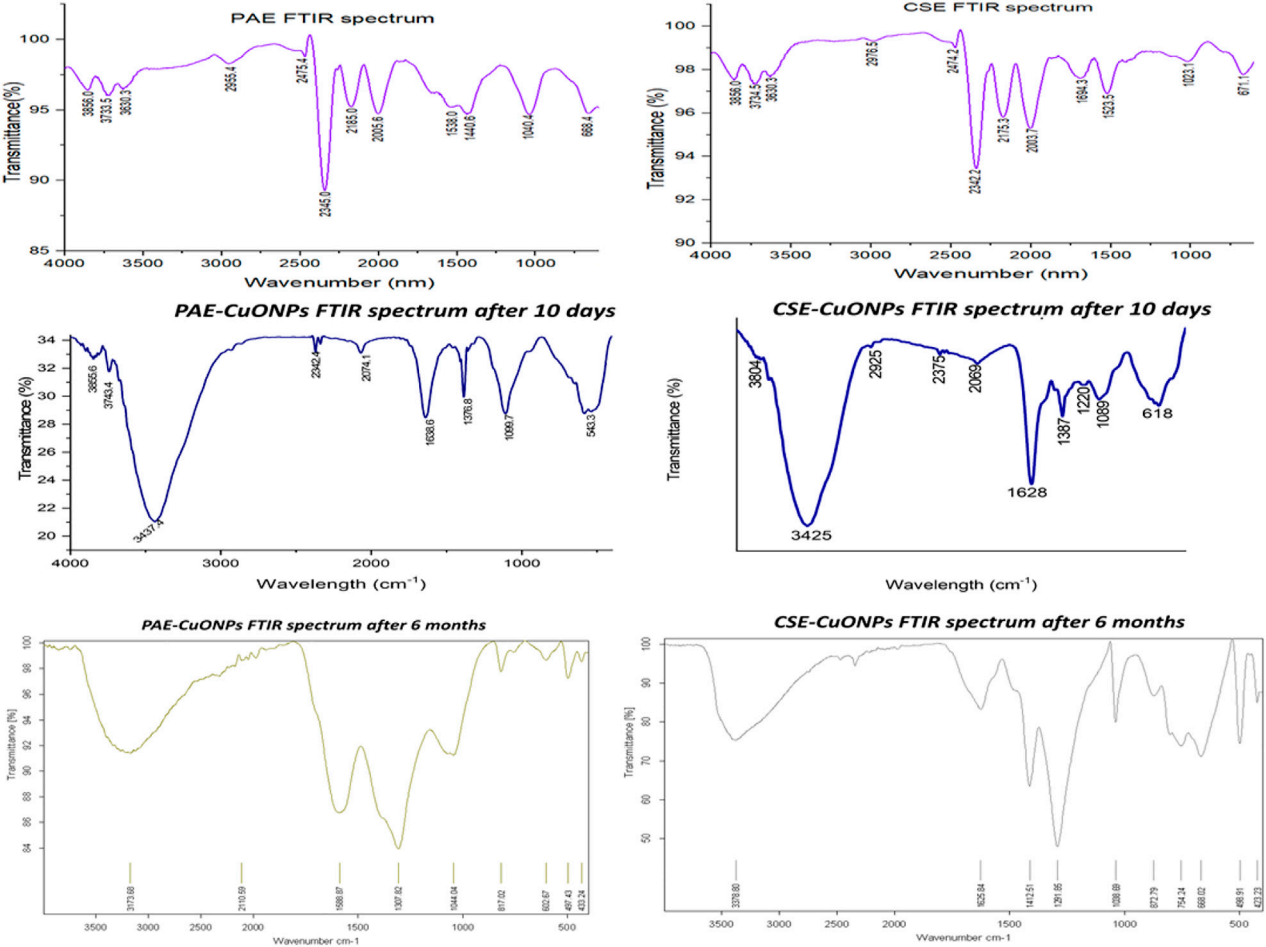
FTIR absorbance spectra for PAE, CSE, PAE-CuONPs, and CSE-CuONPs. PAE stands for Prunus africana extract, CSE; Camellia sinensis extract, PAE-CuONPs; Prunus africana biosynthesized copper oxide nanoparticles and CSE-CuONPs; Camellia sinensis green synthesized copper oxide nanoparticles.

The FTIR spectra of plant extracts, PAE, and CSE green synthesized CuONPs gave similar absorbance bands falling within the wavenumber range of 3,650–3,400, 2,349, 2,140–1990, 1,650–1,600, 1,390–1,370, 1,250–1,020 1,124–1,087, and 690–515 cm^−1^. Copper oxide nanoparticles generated by PAE registered two distinct bands falling within 4,000–3,700 cm^−1,^ whereas CSE biosynthesized CuONPs recorded two unique absorbance bands in the range of 2,960–2,850 and 1,250–1,020 cm^−1^, Table 2. After 6 months of storage, FTIR analysis yielded comparable spectra with freshly prepared CuONPs, Figure 3.

**TABLE 2:** PAE and CSE phytochemical functional groups encapsulation CuONPs.

Peak intensity	PAE wavenumber (cm^−1^)	CSE wavenumber (cm^−1^)	CuONPs-PAE wavenumber (cm^−1^)	CuO NPs-CSE wavenumber (cm^−1^)	Functional group wavenumber range	Bond	Functional group
Low	3,856	3,856	3,847	3,804	4,000–3,700	O-H	Water
Low	3,734	3,734	3,743	—	4,000–3,700	O-H	Water
High	—	—	3,438	3,425	3,650–3,400	0-H	Alcohol
low	3,630	3,630	—	-	3,650–3,400	0-H	Alcohol
Low	2,955	2,977	2,937	2,925	2,960–2,850	C-H	Alkane
Low	2,475	2,474	—	—	2,400–2000	O=C=O	Carbon dioxide
Low	—	—	2,342	2,375	2,400–2000	O=C=O	Carbon dioxide
High	2,345	2,342	—	—	2,400–2000	O=C=O	Carbon dioxide
Low	2,185	2,175	2074	2069	2,140–1990	N=C=S	Isothiocyanate
Medium	2006	2003	—	—	2,140–1990	N=C=S	Isothiocyanate
Medium	—	1,694	1,639	1,628	1,650–1,600	C=C	Conjugated alkene
Medium	1,538	1,524	—	—	1,550–1,500	N-O	Nitro compound
Medium	1,441	—	1,377	1,387	1,390–1,370/1,450	C-H	Alkanes
Low	—	1,233		1,220	1,250–1,020	C-N	Amines
Medium	1,040	1,023	1,100	1,089	1,124–1,087	C-O stretching	Secondary alcohol
Medium	668	671	543.3	618	690–515	C-Br stretching	Alkyl halide

### Dynamic Light Scattering and Zeta Potential

Dynamic light scattering analysis showed that the average size distribution of nanoparticles ranged from 4 to 620 nm with a mean distribution diameter of 61 and 68 nm for CSE and PAE green synthesized CuONPs, respectively, while electrophoretic light scattering charge computation revealed that the zeta potential fluctuated between +11.8 and +13.6 mV with an average charge of +12.5 mV.

### Scanning Electron Microscopy Analysis

**FIGURE 4:**
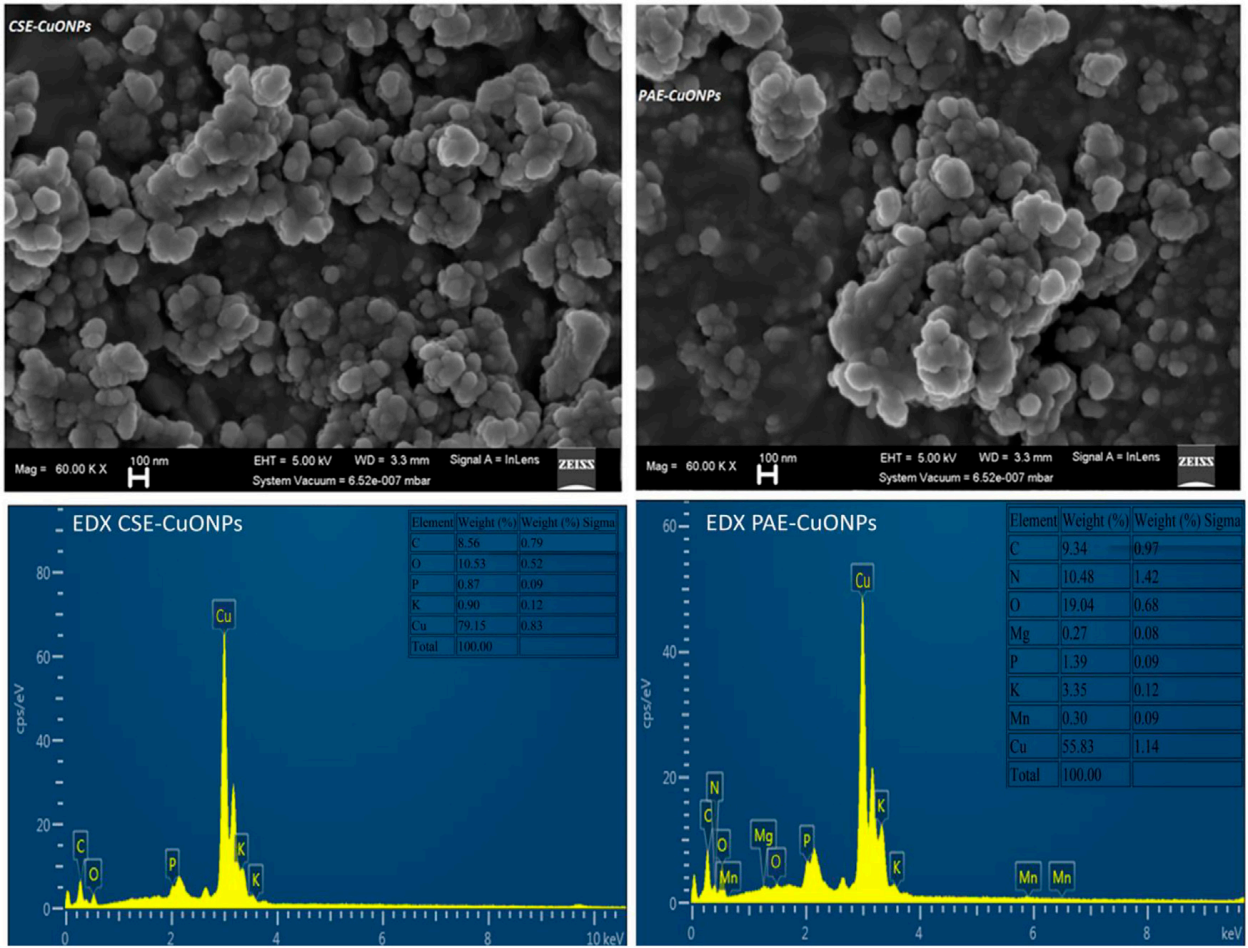
FESEM Micrographs and EDX spectra for green synthesized copper nanoparticles. CSE-CuONPs and PAE-CuONPs represent Camellia sinensis and Prunus africana green synthesized copper nanoparticles, respectively.

Morphology and size of the CuONPs were obtained from the field emission scanning electron microscopy (FESEM) micrographs, Figure 4. The FESEM images exhibited clustered CuONPs with spherical shapes, size distribution ranging from 3 to 192 nm (CuONPs-CSE) and 4–576 nm (CuONPs-PAE), and mean diameter of 6 and 8 nm for CSE and PAE biosynthesized nanoparticles, respectively. Additionally, FESEM images showed that PAE biosynthesized nanoparticles were covered by a layer of viscous material. Elemental analysis by EDX revealed that the samples had copper and oxygen as the main constituents with contaminants such as nitrogen, potassium, phosphorus, magnesium and manganese, Figure 4. Furthermore, the CSE biosynthesized sample contained 79.15% copper and 10.53% oxygen, while PAE derived sample had 55.83% copper and 19.04% oxygen.

### Antibacterial Activity of CuONPs

The antibacterial activity of CuONPs was assessed against both Gram-positive and Gram-negative multidrug-resistant (MDR) bacterial strains by determining the zones of growth inhibition. Compared to standard antibiotics, the efficacy of the CuONPs against the MDR strains was found to be significant (*p* < 0.0001). The diameter of the growth inhibitory zones increased with an increase in the concentration of the CuONPs and was significantly more extensive in MRSA with low MIC and MBC of 30 μg/ml and 125 μg/ml, respectively compared to *E. coli* and *K. pneumoniae*, Tables 3, 4 and Supplementary Figure S1. Statistically, similar growth inhibitory zones were obtained with CuONPs after a six-month storage period, Table 3 and Supplementary Table S2.

**TABLE 3:** MIC and MBC values of biosynthesized CuONPs against Carbapenem-resistant and sensitive *E. coli* and *K. pneumoniae*.

Bacteria type	PAE-CuONPs		CSE-CuONPs	
	MIC (µg/ml)	MBC (µg/ml)	MIC (µg/ml)	MBC (µg/ml)
Carbapenem resistant *E. coli*	125^a^	250^ab^	125^a^	250^ab^
Carbapenem resistant *K. pneumoniae*	125^a^	250^ab^	125^a^	250^ab^
Methicilin reistant *Staphyloccocus aureus*	30^c^	125^d^	30^c^	125^d^

Mean values in each column accompanied by the same letter are not significantly different (*p* > 0.05) (Tukey Multiple Comparison), and values accompanied by letter (s) which are not similar are significantly different (*p* < 0.05). MIC, Minimum inhibitory concentration, MBC, Minimum bactericidal concentration, PAE-CuONPs, *Prunus africana* extract biosynthesized copper oxide nanoparticles and CSE-CuONPs, and *Camellia sinensis* extract biosynthesized copper oxide nanoparticles.

**TABLE 4:** Comparative statistics of antimicrobial activities, size, shape, and zeta potential of copper nanoparticles synthesized by using various plant.

Source of plant extract	Pathogen	ZOI (mm) 250 μg/ml	ZOI (mm) 100 μg/ml	ZOI (mm) 50 μg/ml	Size (nm)	Shape	Zeta potential	References
*Camellia sinensis*	*Escherichia coli*	27	12	6	6	Spherical	+12.5	Current study
	*Klebsiella pneumoniae*	27	11	7				Current study
	*Staphylococcus aureus*	30	20	17				Current study
*Prunus africana*	*Escherichia coli*	26	11	5	8	Spherical	+12.5	Current study
	*Klebsiella pneumoniae*	27	11	4				Current study
	*Staphylococcus aureus*	30	21	17				Current study
*Hagenia abyssinica (Brace) JF. Gmel.*	*Staphylococcus aureus*	15	NA	NA	34	Spherical, hexagonal, triangular, cylindrical, and irregular	—	Murthy et al. (2020)
	*E. coli*	13	NA	NA				
	*Pseudomonas aeruginosa*	13	NA	NA				
	*Bacillus subtilis*	14	NA	NA				
*Cissus vitiginea*	*E. coli*	22	NA	NA	20	Spherical	—	(Wu et al., 2020)
	*Klebsiella pneumoniae*	19	NA	NA				
	*Enterococcus* sp.	20	NA	NA				
	*Proteus sp*	16	NA	NA				
*Momordica charantia*	*E. coli*	25	NA	NA	62	Rod	−7.3	Qamar et al. (2020)
	*Klebsiella pneumoniae*	25	NA	NA				
	*Bacillus cereus*	32	NA	NA				
	*Pseudomonas aeruginosa*	26	NA	NA				
	*Proteus vulgaris*	26	NA	NA				
	*Streptococcus mutans*	29	NA	NA				
	*Staphylococcus aureus*	29	NA	NA				
	*Streptococcus pyogenes*	26	NA	NA				
	*Streptococcus viridians*	27	NA	NA				
	*Staphylococcus epidermidis*	23	NA	NA				
	*Corynebacterium xerosis*	29	NA	NA				
*Ageratum houstonianum Mill*	*E. coli*	12	NA	NA	80	Cubic, hexagonal, and rectangular	—	Chandraker et al. (2020)

### Photocatalytic Degradation of Methylene Blue

**FIGURE 5:**
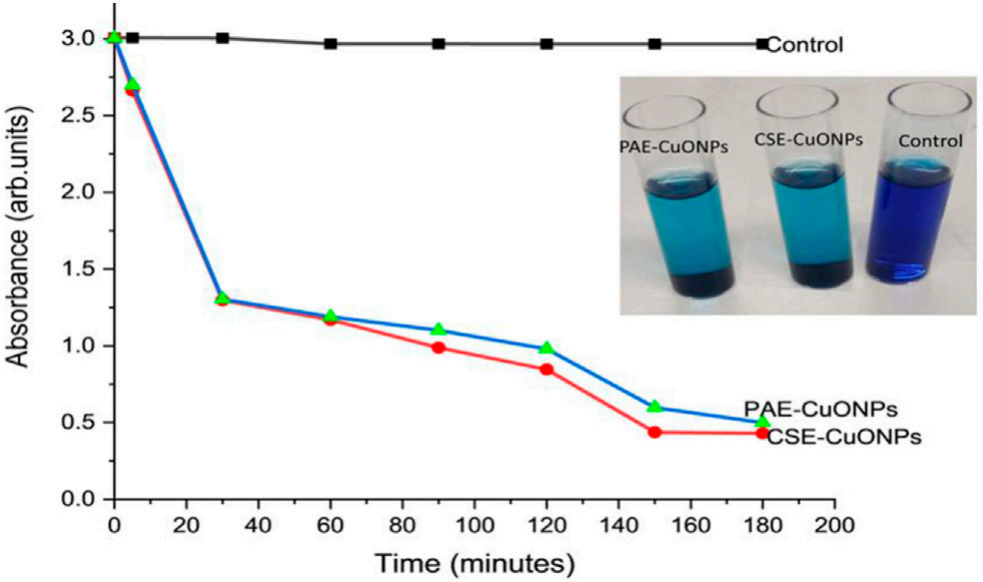
Time-dependent photocatalytic degradation of methylene blue under solar irradiation. Control represents methylene blue, CSE-CuONPs represents methylene blue treated with Camellia sinensis biosynthesized copper oxide nanoparticles, and PAE-CuONPs represents methylene blue treated with Prunus africana biosynthesized copper oxide nanoparticles.

CuONPs mediated photocatalysis of MB under sunlight was time-dependent. The time-dependent reduction in the absorption intensity of MB dye was registered after the treatment with CuONPs under sunlight irradiation, Figure 5. The concentration of MB reduced from 100 μg/ml to 14.5 μg/ml with a degradation efficiency of 85.5% and 16.8 μg/ml (83.2% degradation efficiency) for CSE-CuONPs and PAE-CuONPs photocatalyzed degradation respectively after 180 min. The percentage degradation efficiency of CSE-CuONPs was higher but statistically similar to that of PAE-CuONPs, Table 5. For the setups kept under darkness, no degradation was observed as the absorbance recorded were similar to those of the control.

**TABLE 5:** Concentration of methylene blue and degradation efficiency after CuONPs photocatalysis calculated from absorbance at different time intervals.

Time (minutes)	Control abs (arb.units)	CSE-CuONPs-MB abs (arb.units)	PAE-CuONPs-MB abs (arb.units)	CSE-CuONPs Ct (µg/ml)	PAE-CuONPs Ct (µg/ml)	CSE-CuONPs DE (%)	PAE-CuONPs DE (%)	*p* Value
0	3.006	3.005	3.005	100.0	100.0	0.0	0.0	*p* Value
5	3.006	2.662	2.699	88.6	89.8	11.4	10.2	—
30	3.004	1.297	1.304	43.2	43.4	56.8	56.6	0.9999
60	2.967	1.169	1.189	39.4	40.1	60.6	59.9	0.9997
90	2.967	0.988	1.101	33.3	37.1	66.7	62.9	0.9899
120	2.966	0.846	0.979	28.5	33.0	71.5	67.0	0.9997
150	2.966	0.437	0.598	14.7	20.2	85.3	79.8	0.1237
180	2.966	0.43	0.499	14.5	16.8	85.5	83.2	0.9975

### Antioxidant Activity of the Plant Extracts and Biosynthesized CuONPs

The DPPH assay was used to evaluate the percentage antioxidant activity of the biosynthesized CuONPs and the plant extracts, where ascorbic acid was used as the standard antioxidant. The plant extracts (CSE and PAE) exerted substantial antioxidant activity, while the biosynthesized CuONPs exhibited lower activity, Table 6.

**TABLE 6:** Antioxidant activity percentage of the plant extracts and green synthesized copper nanoparticles.

Concentration of antioxidant	Scavenging activity (%)				
	CSE	PAE	CSE-CuONPs	PAE-CuONPs	Ascorbic acid
50 μg/ml	30.2	22.4	17.5	16.9	23.4
100 μg/ml	43.1	30.9	19.3	19.9	34.6
150 μg/ml	49.9	45.0	23.7	23.8	44.2
200 μg/ml	60.1	51.2	24.2	23.9	54.2
250 μg/ml	68.3	64.1	26.8	25.4	63.9
300 μg/ml	77.5	70.2	28.8	28.5	70.8
Mean	54.9^a^	47.3^a^	23.4^b^	23.1^b^	48.5^a^

Mean values in each column accompanied by the same letter are not significantly different (*p* > 0.05) (Tukey Multiple Comparison), and values accompanied by letter (s) which are not similar are significantly different (*p* < 0.05).

## Discussion

**SCHEME 1:**
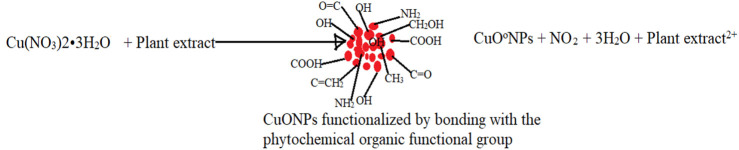
Probable mechanism of bio-reduction of Copper nitrate to functionalized CuONPs

UV Vis spectroscopic analysis confirmed bio-reduction of copper nitrate to CuONPs as surface plasmon resonance (SPR) peaks were registered between 272 and 275 nm for CSE-CuONPs and 281–286 nm for CSE-CuONPs, Scheme 1. The SPR peaks are characteristic of the surface plasmon band of CuONPs. This agrees with several studies which found that UV Vis absorbance of CuONPs fall between 250 and 275 nm (Dutta et al., 2015; Kumar et al., 2017; Berra et al., 2018; Khashan et al., 2018). Furthermore, CSE-mediated bio-reduction of copper nitrate to CuONPs was more pronounced in acidic pH. In contrast, peak intensities revealed that PAE mediated synthesis was highly efficient in slightly acidic (pH 5.43) and alkaline pH. Higher intensity UV Vis absorbance peaks for setup one that involved heating at high temperature may be attributed to highly concentrated CuONPs in solution, indicating that nanoparticles are efficiently green synthesized at higher temperatures. UV Vis absorbance shift from the short wavelength to long wavelength is mainly associated with an increase in nanoparticle size; thus, CSE green synthesized smaller nanoparticles than PAE. All the UV Vis absorbance spectra for the controls (CSE and PAE) and the mixture of plant extracts and Copper II nitrate solutions exhibited absorption peaks between 225 and 230 nm associated with the benzoyl rings. These peaks are related to π ⇾ π transitions demonstrating that polyphenols are some of the constituent phytochemicals in the CSE and PAE (Atarod et al., 2016).

X-Ray Diffraction analysis displayed nine peaks for both CSE and PAE phytosynthesized CuONPs. Bragg peaks positioned at 2θ angles of 35.5°, 38.7°, 48.9°, 53.5°, 58.3°, 61.5°, 68.1°, 72.5°, and 75.3° for both PAE and CSE mediated CuONPs, and these XRD angles correspond to crystal plane/Miller’s indices (002), (111), (202ˉ), (020), (202), (113^-^), (220), (311) and (222^-^) respectively. PAE mediated CuONPs had two unique extra peaks corresponding to 2θ values of 32.5° and 66.3° matching well with crystal plane (110) and (311^-^) while CSE fabricated CuONPs expressed one exceptional peak at 2θ value of 65.9° matching with Miller’s index (022). All the registered cubic crystal lattice planes/Miller’s indices agree with the standard values for copper oxide nanoparticles reported by the International Centre for Diffraction Data (ICDD) database, file number 048–1548. Similar XRD patterns were obtained from both freshly prepared and stored (6 months) nanoparticles indicating that biosynthesized nanoparticles have a long shelf-life. Furthermore, elemental analysis by EDX confirmed copper and oxygen as the predominant constituents of the biosynthesized samples. Contaminant elements such as nitrogen, potassium, phosphorus, magnesium and manganese revealed by EDX might have been contributed by the plant extracts. The CSE biosynthesized sample contained 79.15% copper and 10.53% oxygen with limited impurities, while PAE derived sample had 55.83% copper and 19.04% oxygen with a lot of unexpected elements; thus, elemental analysis shows that CSE efficiently biosynthesized CuONPs.

Fourier Transform Infrared spectrophotometry was used to analyze the plant extract phytochemicals involved in reducing copper ions to CuONPs and subsequent capping. The organic functional groups entrapping CuONPs were assigned according to (Pongpiachan, 2014; Merck (2021).Spectrum, 2021). The FTIR spectra of both plant extracts (PAE and CSE), PAE, and CSE green synthesized CuO NPs registered similar absorbance bands between 3,650–3,400 cm^−1^ associated to the O-H intermolecular stretching bonds of phenolic substance, 2,960–2,850 cm^−1^ band for C-H representing stretching vibrations of methyl functional groups of alkanes, 2,349 cm^−1^ responsible for O=C=O bonds of Carbon dioxide, 2,140–1990 cm^−1^ linked to N=C=S bonds corresponding to isothiocyanate, 1,650–1,600 cm^−1^ related C=C bond of conjugated alkene, 1,390–1,370 cm^−1^ matching C-H bond of alkanes, 1,124–1,087 cm^−1^ allied to C-O stretching secondary alcohol bond and 690–515 cm^−1^ connected to C-Br stretching of alkyl halide. FTIR absorbance spectra for CSE and CSE-CuONPs exhibited one unique band (1,250–1,020 cm^−1^) for the C-N stretching bond of amines. Furthermore, the FTIR absorbance spectrum for PAE, CSE, and PAE-CuONPs had two exceptional bands located between 4,000–3,700 cm^−1^ typical of hydroxy group attributed to water, whereas both PAE and CSE exhibited one exclusive band between 1,550–1,500 cm^−1^ characteristic of N-O stretching bond of nitro compounds*.* The FTIR results indicate the capping of CuONPs by the phytochemicals used in green synthesis. Several studies reported similar findings (Nagar and Devra, 2018; Sharma et al., 2019; Mali et al., 2020). *Prunus africana* phytochemical profiling revealed the presence of pentacyclic triterpenoids that possess terminal OH and COOH groups (Donovan et al., 1998), phytosterols with OH, ethyl, and methyl as the main functional groups (Carbin et al., 1990; Bin Sayeed et al., 2016), and ferulic acid esters terminated by aromatic rings, COOH, -CHO, -CH2OH, -CH3, and -COOC2H5 (Nenadis et al., 2003; Kampa et al., 2004) whereas *Camellia sinesis* processed into green tea extract comprises of polyphenols catechins contains benzene rings with OH and methyl groups (Botten et al., 2015; Takahashi et al., 2018). This corroborates well with the functional groups of PAE and CSE capping CuONPs revealed by FTIR analysis in this study. Furthermore, alkyl halides and Isothiocyanates identified by FTIR in this study as components of PAE and CSE were documented as constituents of PE phytochemical (Palliyaguru et al., 2018; Suganya et al., 2019). Furthermore, the FTIR spectroscopy was performed on the copper oxide nanoparticles 10 days and 6 months after synthesis registered comparable functional groups. This clearly showed that the phytochemicals which capped, stabilized, and functionalized the nanoparticles during synthesis were still present after 6 months. These findings indicate that the biosynthesized CuONPs can be used for different applications after prolonged storage.

FESEM micrograph revealed that the biosynthesized CuONPs were uniformly spherical with some degree of aggregation. The generation of monodisperse nanospheres may be due to homogeneous nucleation during rapid bio-reduction of the copper salt to nanoform (Chaudhary et al., 2018). DLS, XRD, and FESEM estimated the size distribution of copper nanoparticles. The three platforms revealed that CSE fabricated smaller nanoparticles than PAE. However, both CSE and PAE biosynthesized CuONP were spherical in shape. Size influences the chemical reactivity and bioactivity of nanoparticles. Different plant species possess variant phytochemical profiles with considerably different antioxidant activity; thus, the plant extract constituent phytochemicals influence the size and morphology of nanoparticles produced (Mittal et al., 2013). This provides an insight as to why CSE biosynthesized highly concentrated smaller CuONPs. Indeed, phytochemical profiling presented pentacyclic triterpenoids (ursolic and oleanolic acids) (Donovan et al., 1998), phytosterols chiefly *β*-sitosterol and *β*-sitostenone (Carbin et al., 1990; Bin Sayeed et al., 2016), and ferulic acid esters (n-tetracosanol and n-docosanol) (Nenadis et al., 2003; Kampa et al., 2004) as the constituent phytochemicals with antioxidant activity in PAE. Polyphenols catechins with potent antioxidant activity have been reported as the main phytochemicals in *Camellia sinesis* processed into green tea extract (Botten et al., 2015).

Stability and spacing of nanoparticles are mainly achieved when the constituent nanoparticles have a net charge of +30 mV. On the assumption that all the nanoparticles in the pool have the same charge, such a net charge facilitates repelling of the constituent nanoparticles; thus, thwarting clustering and hence maintaining stability (Müller et al., 2001; Ssekatawa et al., 2020). However, the biosynthesized CuONPs registered a net charge of +12.5 mV in this study. Therefore, clustering of the CuONPs revealed by FESEM micrographs may be due to this low net charge.

The biosynthesized CuONPs exhibited potent antibacterial activity in a dose-dependent. Indeed, this corroborates with other studies which reported that the antibacterial activity of CuONPs increases with an increase in concentration (Amin et al., 2021; Andualem et al., 2020). A significantly large inhibition zone (30 mm) and low MIC (30 μg/ml) were observed in *S. aureus,* Gram-positive bacteria. Similar findings were reported by Qamar et al. (Qamar et al., 2020). In contrast, Andualem et al. (Andualem et al., 2020) observed that CuONPs were more efficacious against Gram-negative bacterial cells. Furthermore, comparable zones of bacterial growth inhibition and MIC by biosynthesized Copper nanoparticles were reported by several studies (Wu et al., 2020; Arya et al., 2018; Hassanien et al., 2018). The CuONPs were statistically more efficacious than the standard drugs against MDR bacteria, indicating that they are potential alternatives to antibiotics. The potent antibacterial activity of the phytosynthesized CuONPs may be accredited to the overall positive zeta potential/charge. Bacterial cell membrane exhibits a net negative charge that greatly attracts positively charged materials (Ssekatawa et al., 2020). Due to this electrostatic interaction, adsorption and consequently penetration of bacterial cells by CuONPs is enhanced. Contrary to this, Qamar et al. (Qamar et al., 2020) biosynthesized large rod-shaped copper nanoparticles with a net charge of −7.3 mV and diameter of 62 nm but with potent antimicrobial activity against both gram-positive and gram-negative bacteria, Table 4. The potent antibacterial activity in Qamar et al. (Qamar et al., 2020) was due to the shape of the nanoparticles. Rod-shaped nanoparticles present a large surface area to volume ratio that enhances their interaction with the bacterial cells, thereby inhibiting their growth. This clearly indicates that several parameters influence the antimicrobial activity of nanoparticles. Furthermore, the synthesis of inorganic nanoparticles with a range of morphologies affords them excellent antimicrobial activity as each morphology presents a unique mechanism of bactericidal action. Contrary to this, CuONPs generated by this study were all spherical and exhibited potent antibacterial activity. This is in line with other studies that green synthesized uniformly shaped copper nanoparticles (Wu et al., 2020; Qamar et al., 2020; Siddiqi et al., 2020). Moreover, studies that biosynthesis polymorphic copper nanoparticles reported lower MICs (Chandraker et al., 2020; Murthy et al., 2020), Table 4. To assess the impact of the storage time on the antibacterial activity of the CuONPs, the agar well diffusion assay was repeated with nanoparticles stored for 6 months at room temperature. Freshly prepared and six-month-old CuONPs exhibited statistically similar antibacterial activity, indicating that the biosynthesized nanoparticles were highly stable and could be used after prolonged storage. Similar findings were reported by Qamar et al. (Qamar et al., 2020)

The potential of the green synthesized CuONPs to photocatalyzed the degradation of MB was examined under direct sunlight. The optimal pH for photodegradation of dyes in the presence of metal nanoparticle photocatalyst basic (pH = 9). The surface charge properties of the photocatalyst are influenced by the alkaline pH. The negatively charged anionic dye binds on the photocatalyst surface during photocatalysis, and alkaline pH facilitates the adsorption process (Mali et al., 2020). To investigate that dye degradation is catalyzed metal nanoparticles under high-intensity sunlight, the experiments were carried out in the presence and absence of catalyst with exposure to light and darkness. CuONPs presented no significant photocatalytic activity under darkness. Furthermore, degradation of MB dye was achieved under direct sunlight in the presence of CuONPs catalyst. Whereas the setup without the catalyst exhibited insignificant dye degradation. Thus, the photocatalysis assay portrayed that a photocatalytic process influences dye degradation. In contrast, no degradation of MB was observed when Chandraker et al. (Chandraker et al., 2020) used copper nanoparticles as photocatalysts. Furthermore, both CSE and PAE biosynthesized nanoparticles demonstrated statistically similar photocatalysis activity.

Several human metabolic processes yield free radicals. The free radicals are unstable and cause cellular damage due to the liberation of reactive oxygen species that interact with molecules in other unrelated biochemical pathways. Thus, free radicals are responsible for many degenerative disorders and reduced immunity against infections. Antioxidants prevent the disorders by scavenging oxygen-derived free radicals through donating or accepting electrons from the rich reactive oxygen species (Liu et al., 2018). Medicinal plants containing phenolic compounds are used as antioxidants to neutralize the free radicals. The antioxidant activity of the biosynthesized CuONPs against DPPH radical was evaluated and compared with CSE, PAE, and the standard ascorbic acid. The percentage scavenging activity of green synthesized CuONPs increased with increasing CuONPs concentration and in line with the plant extracts and the standard. Therefore, the maximum antioxidant activity was registered at the highest concentration of the antioxidants used. However, the standard and plant extracts recorded significantly high percentage scavenging activity. Similar findings were reported by Das et al. (Das et al., 2020). Indeed, ascorbic acid is a certified antioxidant, whereas *Camellia sinensis* and *Prunus africana* contain polyphenols and other phytochemicals with potent antioxidant activity and are used are natural antioxidants to manage degenerative diseases (Ssekatawa et al., 2021c). Furthermore, the mechanism behind the antioxidant activity of inorganic nanoparticles is attributed to the binding of transition metal ion catalysts by the free radicals hence enhancing the radical scavenging activity. The CSE and PAE exhibited significantly high antioxidant activity. FTIR analysis confirmed the capping of the CuONPs with the phytochemicals used for their biosynthesis. The antioxidant activity may be enhanced by the various bio-reductive groups of the phytochemicals capping the CuONPs.

The physicochemical properties and performance of the CuONPs were based on to compare the robustness of CSE and PAE to biosynthesize inorganic nanoparticles. UV Vis spectroscopy, DLS, XRD, and FESEM micrographs revealed that CSE synthesized smaller CuONPs than PAE; however, the CuONPs biosynthesized by both the plant extracts exhibited statistically similar MICs, photocatalytic and antioxidant activities. Therefore, CSE-CuONPs and PAE-CUONPs displayed identical performances. However, FESEM and elemental analysis revealed that PAE-CuONPs had a high percentage of contaminants.

## Conclusion

Emergence of antibiotic-resistant strains has dramatically affected the healthcare systems worldwide. Several studies have endorsed nanomaterials as alternatives or potentiators of antibiotics as carriers. However, traditional methods used to synthesize inorganic nanoparticles have limitations that have affected their application in settings with limited resources. Thus, the use of green chemistry approaches by exploiting phytochemicals with antioxidant activity enables the economical, eco-friendly and simple process for the synthesis of nanoparticles. Therefore, this study successfully used *Prunus africana* and *Camellia sinensis* extracts to biosynthesize functionalized CuONPs. XRD analysis and UV Vis spectroscopy exhibited patterns characteristic to CuONPs. XRD pattern and FTIR analyses confirmed that the CuONPs remained crystalline and functionalized by phytochemical functional groups after 6 months of storage. The biosynthesized CuONPs had a mean diameter of 6 nm with a net charge of +12.5 mV and possessed potent antibacterial activity against MRSA, carbapenem-resistant *E. coli* and *K. pneumoniae* at low MICs. Furthermore, the CuONPs exhibited excellent photocatalytic and antioxidant activities. In the light of potent antibacterial activity shown by the biosynthesized CuONPs in this study, we are currently exploring the possibility of re-potentiating antibiotics by designing copper nanoparticle-based drug delivery systems. This will involve conjugating the functionalized CuONPs with antibiotics and evaluation of the combinatory effects of the complex against MDR bacteria. Furthermore, one of the drivers of antimicrobial resistance is the formation of biofilms on indwelling biomedical devices. Thus, we intend to design bioactive catheters with antimicrobial activity by coating them with thin films of biosynthesized CuONPs. With the potent antimicrobial, photocatalytic and antioxidant activities, biosynthesized CuONPs have the potential to be used in several biomedical and industrial applications.

## Data Availability

The original contributions presented in the study are included in the article/Supplementary Material, further inquiries can be directed to the corresponding author.
