# Supplementary material for: Phyto-Mediated Copper Oxide Nanoparticles for Antibacterial, Antioxidant and Photocatalytic Performances
Source: Front Bioeng Biotechnol. 2022 Feb 16;10:820218. doi: 10.3389/fbioe.2022.820218 (PMC8889028; doi:10.3389/fbioe.2022.820218)
Supplement: Supplementary file 1 [file DataSheet1.PDF]

Plant extracts mediated green synthesis and characterization of copper oxide nanoparticles and the study of their antibacterial, photocatalytic and antioxidant activities

Kenneth Ssekatawa[1], Denis K Byarugaba[1], Martin Kamilo Angwe[1], Eddie M. Wampande[1], Francis Ejobi[1], Edward Nxumalo[5], Malik Maaza[3&4], Juliet Sackey [3&4] and John Baptist Kirabira[2]

1. College of Veterinary Medicine Animal Resources and Biosecurity, Makerere University P. O. Box 7062 Kampala Uganda; *Kssekatee@gmail.com*, *dk@covab.mak.ac.ug*, *martinangwe@gmail.com,* *wampande@yahoo.co.uk* , *ejbifrancis@gmail.com*
2. Africa Center of Excellence in Materials, Product Development and Nanotechnology, College of Engineering, Design, Art and Technology, Makerere University P. O. Box 7062, Kampala Uganda; kirabirajb@gmail.com
3. Nanosciences African Network (NANOAFNET), iThemba LABS-National Research Foundation, Old Faure Road, 7129 Somerset West, South Africa; maaza@tlabs.ac.za, jsackeyunisa@gmail.com
4. UNESCO-UNISA Africa Chair in Nanosciences/Nanotechnology, College of Graduate Studies, University of South Africa (UNISA), Muckleneuk Ridge, PO Box 392, Pretoria, South Africa; maaza@tlabs.ac.za, jsackeyunisa@gmail.com
5. Institute for Nanotechnology and Water Sustainability, College of Science, Engineering and Technology, University of South Africa, Florida Science Campus, 1710, South Africa; nxumaen@unisa.ac.za

Corresponding Author: Prof. John Baptist Kirabira Email: kirabirajb@gmail.com; Tel +256-772 422 738

Table SM1: UV Vis spectra absorbance peaks wavelength and intensities of copper oxide nanoparticles green synthesized at different pH values

	**CSE biosynthesized CuONPs**				**PAE biosynthesized CuONPS**			
**pH**	**Wavelength (nm) at 80º C**	**Intensity (arb.units)**	**Wavelength (nm) at 26º C**	**Intensity (arb.units)**	**Wavelength (nm) at 80º C**	**Intensity (arb.units)**	**Wavelength (nm) at 26º C**	**Intensity (arb.units)**
2	272	1.68	274	1.81	282	0.92	283	1.1
5.43	-	-	-	-	281	2.11	282	1.66
5.62	273	2.43	275	1.0	-	-	-	-
7	273	1.34	274	1.24	283	1.54	285	1.27
9	273	0.98	274	1.39	283	2.14	286	1.29

Table SM2: Sensitivity tests of CuONPs green synthesized by Prunus africana and Camellia Sinensis extracts showing inhibitory zone in millimeters

**Organism**	**Distilled water inhibitory zone (mm)**	**Imipenem disk inhibitory zone (mm)**	**Ampicillin disk inhibitory zone (mm)**	**Inhibitory zone (mm) of 100 µg/mL**			
				**PAE-CuONPs**		**CSE-CuONPS**	
				**Fresh**	**6 months**	**Fresh**	**6 months**
Carbapenem resistant *E. coli*	0^{a}	3^{b}	0^{a}	17^{c}	18^{c}	18^{c}	17^{c}
Carbapenem sensitive *E. coli*	0^{a}	40^{e}	0^{a}	16.5^{c}	17^{c}	19^{c}	17.5^{c}
Carbapenem resistant *K. pneumoniae*	0^{a}	5^{b}	0^{a}	17^{c}	16.5^{c}	18^{c}	17.5^{c}
Carbapenem sensitive *K. pneumoniae*	0^{a}	40^{e}	0^{a}	17^{c}	17^{c}	18^{c}	18^{c}
Methicillin resistant *S. aureus*	0^{a}	29^{d}	0^{a}	21^{c}	20^{c}	21^{c}	20^{c}

Mean values in each column accompanied by the same letter are not significantly different ($P > 0.05$) (Tukey Multiple Comparison) and values accompanied by letter (s) which are not similar are significantly different (P <

0.05). PAE-CuONPs: *Prunus africana* extract biosynthesized copper oxide nanoparticles and CSE-CuONPs: *Camellia sinensis* extract biosynthesized copper oxide nanoparticles

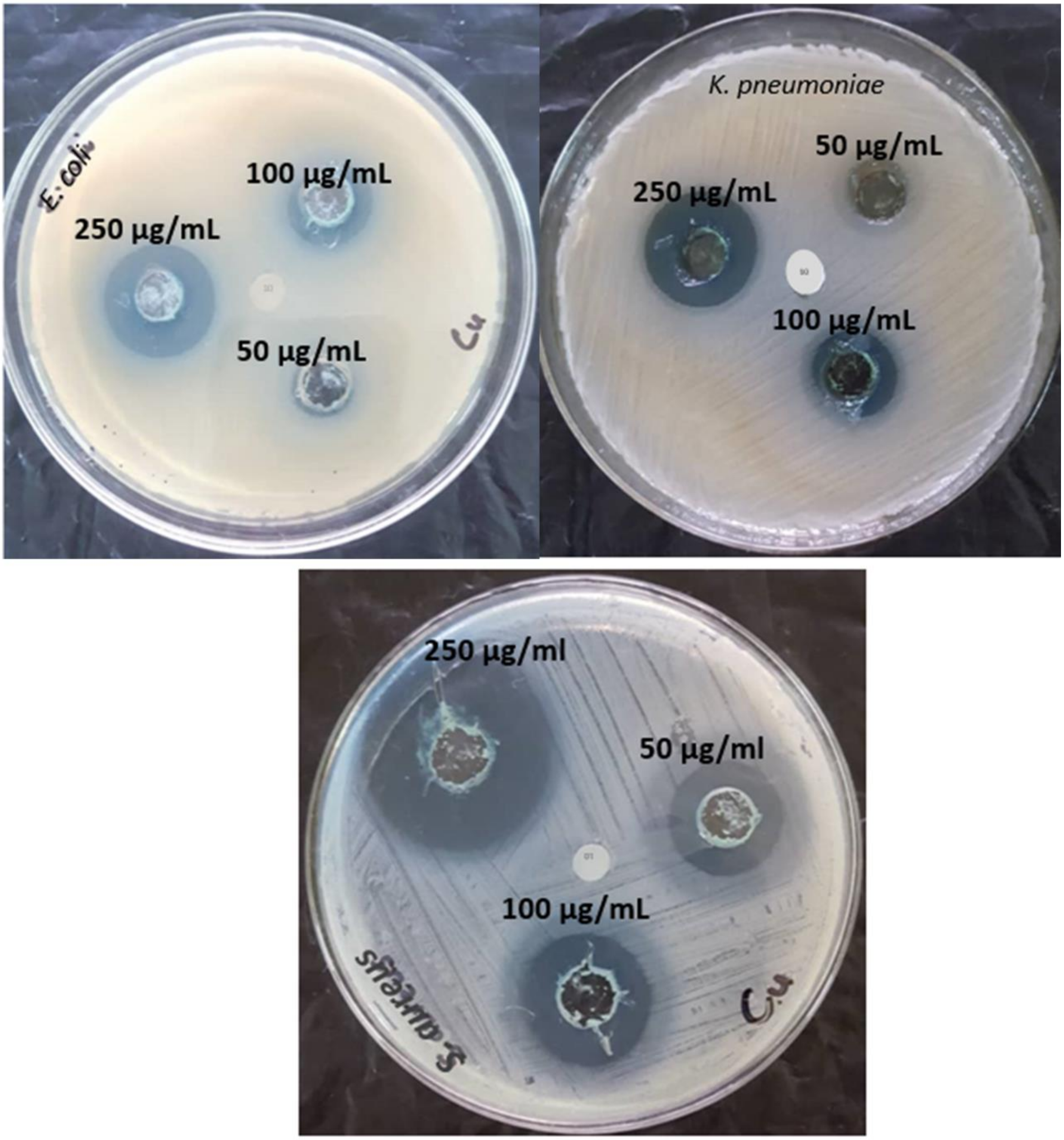

Fig S1: Inhibitory activity of the Copper oxide nanoparticles against carbapenem-resistant *E. coli, K. pneumoniae* and Methicillin-resistant *S. aureus*
